# Disparities in Glycemic Outcomes Persist in Youth with Type 1 Diabetes and High-Technology Use

**DOI:** 10.1155/2023/6646582

**Published:** 2023-10-25

**Authors:** Meryl C. Nath, Blake Frey, Joycelyn Atchison, Jessica A. Schmitt

**Affiliations:** ^1^UAB Heersink School of Medicine, Birmingham, AL 35233, USA; ^2^Department of Pediatrics, University of Alabama at Birmingham, Birmingham, AL 35233, USA

## Abstract

**Background:**

Racial disparities are well described in glycemic outcomes in youth with Type 1 diabetes mellites (T1D). Hemoglobin A1c (HbA1c) has some limitations in comparing glycemia across patient groups as there are individual variations in mean glucose and HbA1c.

**Objective:**

This study aimed to compare glycemic metrics obtained from (Dexcom G6) continuous glucose monitor (CGM) device with HbA1c levels controlling for race, age, duration of diabetes, race, insurance status, and insulin pump use with glycemic control. *Subjects and Methods*. Data analyzed included 188 patients, majority non-Hispanic White (NHW) (*n* = 147, 78.2%) and majority privately insured (*n* = 147, 78.2%). Half of the patients were using insulin pumps, (*n* = 94, 50.0%) and approximately half were female. Median age was 16.6 (interquartile range: 14.2–18.2) years old with a median age of diabetes diagnosis at 9.3-years old.

**Results:**

Significant differences were observed between NHW and non-Hispanic Black (NHB) patients in terms of HbA1c, 90-day mean glucose, and 90-day time >250 mg/dL (>13.9 mmol/L) (7.6% vs. 9.2%, 181 mg/dL vs. 220 mg/dL, and 16.3% vs. 34.7%, respectively, *p* < 0.001 for all comparisons). Multiple linear regression analysis was performed to predict the influence of age, duration of diabetes, race, insurance status, and insulin administration on glycemic outcomes. Regression analysis revealed significant equations for all glycemic outcomes, demonstrating a strong correlation (*p* < 0.0001, *p*=0.0001, and *p* < 0.0001, respectively). However, after controlling for these variables, only race and duration of diabetes remained independently associated with glycemic outcomes, suggesting that these factors strongly influence glycemic control independent of age, sex, insurance, and pump use.

**Conclusion:**

Even in a subset of youth with T1D using CGM with high rates of insulin pump use, disparities in glycemic outcomes persist. When evaluating glycemic outcomes, race remained a significant cofactor despite controlling for age, duration of diabetes, sex, insurance status, and insulin administration type. These results add to the existing literature, and demonstrate race remains strong predictor of glycemic outcomes.

## 1. Introduction

The correlation of persistent hyperglycemia and diabetes complications is well-established [1, 2]. Therefore, major clinical focus is directed toward evaluating and improving glycemic control to reduce the likelihood of developing diabetes-related complications. Evaluation of glycemic control includes measurement of hemoglobin A1c (HbA1c) and self-monitoring of glucose, which can be obtained through fingerstick blood glucose measurements (FSBG) or continuous glucose monitor (CGM) devices. Regarding monitoring glycemic control, CGM has several advantages, including the ability to quantify the proportion of time spent in specific glucose ranges as well as calculate a mean glucose.

Prior to CGM, HbA1c represented the primary mode of assessing long-term glycemic control. However, when describing differences in HbA1c by race, the question of intrinsic differences in rate of glycosylation in different races could complicate the analysis [3]. Namely, are the differences in racial outcomes by HbA1c partially explained by differences in glycosylation rates? Several studies have found significantly higher HbA1c values for Black patients at similar mean glucose measurements compared to White patients [4, 5]. This prior work is limited however in including only mean of FSBG [4] or 14-day CGM data [5].

Additionally, use of diabetes-related technology such as CGM [6–8] and insulin pumps [8] are independently associated with improved glycemic control. Recent work showed that performance of six key diabetes habits, including using an insulin pump and using a CGM or checking FSBG 4+ times per day attenuated the impact of race and socioeconomic status on glycemic outcomes (both HbA1c and time in target range on CGM) [9]. Others have found that the racial disparity in glycemic outcome persists when controlling for income, insurance, and parent education level [3].

Our clinic has made concerted efforts to provide diabetes technology in an equitable way to all patients [10]. As such, we aimed to evaluate if disparities in glycemic control as assessed by HbA1c and 90-day CGM metrics persisted in a population of patients with high rates of CGM and insulin pump use.

## 2. Materials and Methods

This institutional review board-approved review of patients with Type 1 diabetes (T1D) using the Dexcom CGM included glycemic data from HbA1c and corresponding 90-day CGM data, specifically 90-day mean glucose, and 90-day time >13.9 mmol/L (>250 mg/dL). These CGM metrics were selected to best capture both average glycemic value for patients as well as proportion of the day spent with significant hyperglycemia over 13.9 mmol/L (>250 mg/dL). Additionally, 90-day time >13.9 mmol/L (>250 mg/dL) has been shown to be one of the CGM metrics best correlated with HbA1c in those with measured HbA1c of 58 to 80 mmol/mol (7.5%–9.5%) [11].

Subjects were identified by ICD10 codes consistent with T1D (E10.xx) and continuous glucose monitor use as identified by an active prescription. A random selection of 205 patients were selected from this list. Non-CGM data included: race/ethnicity (non-Hispanic White (NHW) and non-Hispanic Black (NHB)), age, insurance (publicly insured and privately insured), age at diagnosis of T1D, sex, duration of diabetes, and insulin pump use. Continuous variables were assessed for normality with D'Agostino and Pearson test. Skewed variables were defined with median and interquartile range (IQR). Paired comparisons of normally distributed variables were done with student *t*-tests with skewed variables compared with Mann–Whitney test. Categorical variables were assessed with Chi-square. Regression models were estimated with binary indicators for race/ethnicity (NHW and NHB), insulin administration (pump vs. injections), and insurance status (public and private). Due to data limitations, type of pump and use of an automated insulin delivery system was unavailable. Analysis was performed in GraphPad Prism 9.1.0®. Due to multiple comparisons, an alpha of 0.01 was set for the statistical significance.

## 3. Results

### 3.1. Subjects

A total of 205 eligible patients were initially identified. Sixteen were removed due to lack of clear documentation on type of insulin administration and one was removed for not having 90-day CGM data available. A total of 188 patients were included in the analysis. Subjects were majority NHW (*n* = 147 (78.2%) and privately insured (*n* = 147, 78.2%). Half of subjects were using insulin pumps (*n* = 94, 50.0%) and approximately half were female (*n* = 89, 47.3%). Subjects had been diagnosed with diabetes at a median of 9.3-years old (IQR: 5.7–12.5) and had diabetes for a median of 5.7 years (IQR: 2.8–10.2). Median age at HbA1c measurement was 16.6 years (IQR: 14.2–18.2).

Comparing patients by race/ethnicity and insurance status, demographics were similar except for current age. Publicly insured patients had a median age of 14.6 years (IQR: 13.2–17.1) compared to privately insured patients who had a median age of 16.9 years (IQR: 14.7–18.5). Race/ethnicity was also associated with insurance status, with 15.0% of NHW patients having public insurance compared to 46.3% of NHB patients (*p* < 0.0001). No difference in frequency of insulin pump use was seen when comparing NHW and NHB patients race (52.4% vs. 41.5%, *p*=0.22) nor was a difference in insulin pump use seen when comparing private and publicly insured patients (52.4% vs. 41.5%, *p*=0.22) (Table 1).

### 3.2. Glycemic Outcomes

Comparisons of HbA1c, 90-day mean glucose, and 90-day time >250 mg/dL (>13.9 mmol/L) for NHW and NHB patients showed significant differences for all metrics (*p* < 0.0001, 0.0005, and 0.0003, respectively). Using an alpha of 0.01, HbA1c in private vs. publicly insured patients were similar (*p*=0.02). HbA1c in private vs. publicly insured subjects was: 7.6% (IQR: 7.0%–8.9%) vs. 8.6% (IQR: 7.6%–9.7%) (59.6 mmol/mol (IQR: 53.0–73.8 mmol/mol) vs. 70.5 mmol/mol (IQR: 59.6–82.5 mmol/mol)). While HbA1c was similar in both insurance groups, CGM glycemic metrics differed.

Privately insured patients had a median 90-day mean glucose of 181 mg/dL (IQR: 162–217 mg/dL) (10.0 (IQR: 9.0–12.0 mmol/L) compared to a median of 218 (IQR: 186–239 mg/dL) (12.1 (IQR: 10.3–13.3 mmol/L)) for publicly insured patients (*p*=0.0013). Similar differences were seen in 90-day time >250 mg/dL (>13.9 mmol/L) (*p*=0.0016) (Table 2).

**Figure 1:**
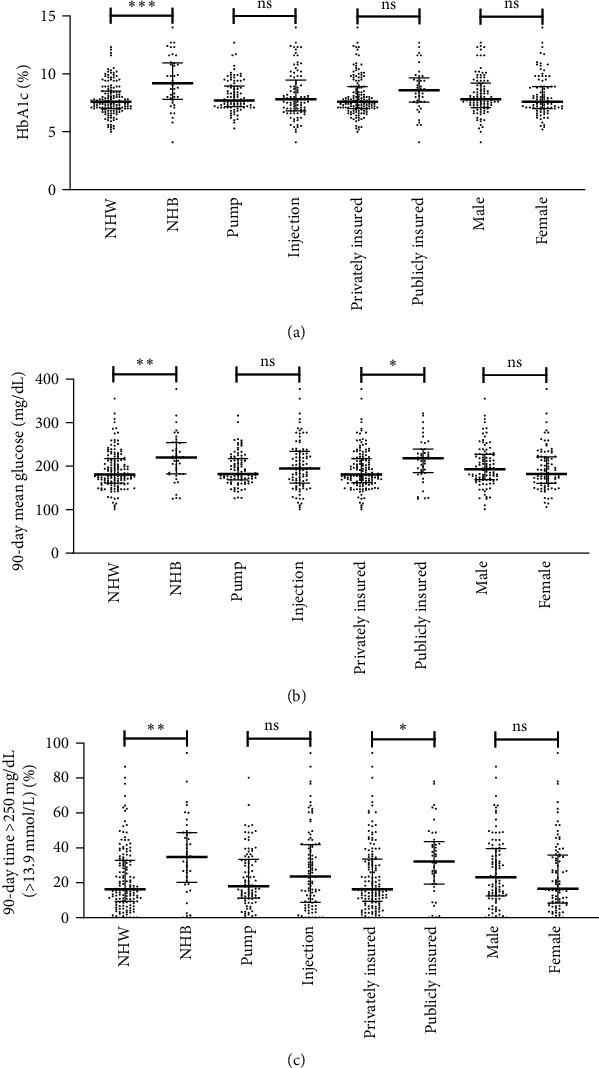
Gylcemic outcomes by race, insulin administration, insurance type, and sex. Median with interquartile range shown (a) HbA1c in % (to convert to mmol/mol substarct 2.15 and multiply by 10.929), (b) 90-day mean glucose in mg/dL (to convert to mmol/L multiply by 0.0555), and (c) 90-day time >250 mg/dL (>13.9 mmol/L) as % of total day ns = not significant,  ^*∗*^*p* < 0.01,  ^*∗∗*^*p* < 0.001, and  ^*∗∗∗*^*p* < 0.0001.

No difference was seen in HbA1c, 90-day mean, or 90-day time >250 mg/dL (>13.9 mmol/L) in those using multiple daily injections compared to those using insulin pumps no were differences in glycemic outcomes seen by patient sex (Table 2 and Figure 1).

A multiple linear regression was calculated to predict glycemic outcomes (HbA1c, 90-day mean glucose, and 90-day time >250 mg/dL (>13.9 mmol/L)) based on current age, duration of diabetes, race, insurance status, and insulin administration type (reference values = privately insured, NHW, pump use, male). The intercept represents the predicted baseline value of the dependent variables (HbA1c (%), 90-day mean glucose (mg/dL), and 90-day time)in our regression model when all independent variables are set to the reference value (for categorical variables) or zero (for continuous variables). Significant regression equations were found for all glycemic outcomes (*p* < 0.0001, *p*=0.0001, and *p* < 0.0001, respectively). Controlling for these variables, only duration of diabetes and race were independently associated with glycemic outcomes (Table 3). Independent of insurance status, sex, and type of insulin administration, NHW patients had improved glycemic metrics relative to NHB patients. While current age was not associated with glycemic outcomes, duration of diabetes was associated, with longer duration of diabetes being associated with poorer glycemic outcomes. Based on our model F, our independent variables explain a significant portion of the variance associated with the dependent variables (HbA1c, 90-day mean glucose, and 90-day time >250 mg/dL). Furthermore, our *R*^2^ value suggests that the independent variables in this regression model (current age, duration of diabetes, race/ethnicity, sex, insurance status, and insulin administration type) are responsible for 18%, 14%, and 15% of the variance in our dependent variables (HbA1c, 90-day mean glucose, and 90-day time >250 mg/dL (>13.9 mmol/L)), respectively.

## 4. Conclusions

Disparities in glycemic outcomes in youth with T1D are well-described [3, 8, 12] and our findings are consistent with this prior work. We add to the existing literature [3, 9] by including 90-day CGM metrics in addition to HbA1c as a marker of glycemic outcomes, mitigating the question of if disparities in glycemic outcomes due to differences in glycosylation rates of HbA1c and/or disparities in access to diabetes technology by race. To our knowledge, this is the first time 90-day CGM data have been utilized in this manner. Furthermore, the inclusion of a large proportion of patients (*n* = 94 (50.0%)) using insulin pumps allowed us to account for the impact of diabetic technology use on glycemic outcome.

By including a high proportion of patients using insulin pumps, we are also able to account for the impact of technology use on glycemic outcome, as both insulin pump use and CGM use are associated with improved glycemic management [6–9]. Unfortunately, we found that when comparing glycemic outcomes, race remained a significant cofactor even when controlling for current age, duration of diabetes, sex, insurance status, and type of insulin administration. Interestingly, duration of diabetes was independently associated with glycemic outcomes independent of current age, increased duration of diabetes was associated with increased HbA1c, increased 90-day mean glucose, and increased time >13.9 mmol/L (>250 mg/dL).

Our cohort had a high proportion (50.0%) of patients using an insulin pump. We saw no difference in frequency of insulin pump use by race or insurance status, suggesting that efforts to reduce disparities in this clinic have been successful. Our data suggest that even in a subpopulation of patients with high access to and use of diabetes-related technologies, NHB patients are at risk for inadequate glycemic control, particularly when compared to their NHW counterparts.

While our cohort of patients using an insulin pump did not have a significant difference in glycemic control relative to those treated with multiple daily injections, it is possible that the lack of difference is reflective of the cross-sectional nature of this project. It is possible that for those currently using insulin pumps, their CGM and HbA1c might have improved relative to when they used multiple daily injections. Additionally, due to the manner in which data were collected, we were unable to distinguish between insulin pumps with and without automated insulin delivery systems. Prospective studies evaluating CGM and HbA1c data for those moving from MDI to insulin pumps and automated insulin delivery systems are needed to further evaluate the impact of diabetes technology in real world settings. Evaluating if socioeconomic cofactors impact the effectiveness of diabetes technologies requires additional focus to ensure patients have equitable access to tools that may improve glycemic control.

Our analysis is limited in that we use insurance status as a proxy for socioeconomic status. With *R*^2^ values of 0.15–0.18 for our models, there are clearly additional factors impacting glycemic control. We are unable to account for household income, caregiver education level, single-parent households, frequency of clinic visits, patient and caregiver literacy/numeracy status, and additional factors that would impact glycemic management. Continued efforts to improve long-term glycemic control in youth with T1D are essential. Diabetes-related technologies have been associated with improved glycemic control, but ideally personalized treatment regimens and support beyond technology can be delivered to at-risk populations and reduce disparities in outcomes.

## Figures and Tables

**Table 1:** Baseline analysis of demographics, diabetes history, and current diabetes management by race and insurance status.

	Total *n* = 188	NHW *n* = 147	NHB *n* = 41	*p*-Value	Privately insured *n* = 147	Publicly insured *n* = 41	*p*-Value
Female	89 (47.3)	68 (46.3)	21 (51.2)	0.57	73 (49.7)	16 (39.0)	0.23
Publicly insured	41 (21.8)	22 (15.0)	19 (46.3)	<0.0001	–	–	–
Current Age (years)	16.6 (14.2–18.2)	16.5 (14.2–18.0)	17.2 (13.6–18.6)	0.69	16.9 (14.7–18.5)	14.6 (13.2–17.1)	0.0003
Age at diagnosis (years)	9.3 (5.7–12.5)	8.8 (5.8–12.6)	9.9 (5.7–12.1)	0.66	9.8 (5.8–12.6)	8.8 (5.5–12.3)	0.46
Diabetes duration (years)	5.7 (2.8–10.2)	5.8 (2.7–10.3)	5.3 (2.8–10.1)	0.92	6.0 (2.8–10.4)	4.9 (1.9–8.5)	0.11
Pump users	94 (50.0)	77 (52.4)	17 (41.5)	0.22	77 (52.4)	17 (41.5)	0.22

NHW, non-Hispanic White; NHB, non-Hispanic Black. Data are expressed in *n* (%) or median (interquartile range) unless otherwise noted.

**Table 2:** Glycemic variability by race, insurance status, insulin administration, and sex.

	Total *n* = 188	NHW *n* = 147	NHB *n* = 41	*p*-Value	Privately insured *n* = 147	Publicly insured *n* = 41	*p*-Value
HbA1c (%)	7.7 (7.1–9.2)	7.6 (7.0–8.5)	9.2 (7.8–11.0)	<0.0001	7.6 (7.0–8.9)	8.6 (7.6–9.7)	0.02
90-Day mean (mg/dL)	190 (166–224)	181 (161–217)	220 (182–254)	0.0005	181 (162–217)	218 (186–239)	0.0013
90-Day time >250 mg/dL (>13.9 mmol/L)	20.0 (10.6–97.3)	16.3 (9.3–32.9)	34.7 (20.2–48.8)	0.0003	16.3 (9.3–33.5)	32.2 (19.2–43.5)	0.0016
	Total *n* = 188	Pump *n* = 94	Injection *n* = 94	*p*-Value	Male *n* = 99	Female *n* = 89	*p*-Value
HbA1c	7.7 (7.1–9.2)	7.7 (7.2–8.9)	7.8 (6.8–9.5)	0.69	7.8 (7.1–9.2)	7.6 (7.0–8.9)	0.51
90-Day mean (mg/dL)	190 (166–224)	182 (169–217)	195 (161–234)	0.39	193 (169–227)	182 (161–222)	0.22
90-Day time >250 mg/dL (>13.9 mmol/L)	20.0 (10.6–97.3)	18.1 (11.2–33.4)	23.7 (8.7–41.9)	0.34	23.1 (12.5–39.5)	16.5 (8.4–35.8)	0.23

NHW, Non-Hispanic White; NHB, Non-Hispanic Black; HbA1c = hemoglobin A1c (in %, to convert to mmol/mol subtract 2.15 and multiply by 10.929); 90-day mean glucose expressed in mg/dL, to convert to mmol/L multiply by 0.0555).

**Table 3:** Results from least square regressions of glycemic outcome, insurance status, race, sex, insulin administration, age, and duration of diabetes.

	HbA1c (%)	90-Day mean glucose (mg/dL)	90-Day time >250 mg/dL (>13.9 mmol/L)
Intercept ^*∗*^	7.48 (6.27–8.69) ^*∗∗∗∗*^	196.3 (162.5–230.2) ^*∗∗∗∗*^	23.86 (9.86–37.87) ^*∗∗∗*^
Insurance (reference: private) ^*∗*^	0.16 (−0.45–0.76)	13.59 (−3.38–30.56)	5.72 (−1.30–12.74)
Race/ethnicity (reference: NHW) ^*∗*^	1.47 (0.88–2.06) ^*∗∗∗∗*^	23.82 (7.37–40.27) ^*∗∗*^	10.88 (4.08–17.69) ^*∗∗*^
Sex (reference: male) ^*∗*^	−0.20 (−0.67–0.27)	−9.67 (−22.73–3.40)	−3.78 (−9.18–1.63)
Insulin administration (reference: pump) ^*∗*^	0.28 (−0.21– 0.77)	12.90 (−0.97–26.77)	6.66 (0.92–12.39)
Current age ^*∗*^	−0.02 (−0.10–0.05)	−1.77 (−3.86–0.33)	−0.72 (−1.58–0.15)
Duration of diabetes ^*∗*^	0.09 (0.04–0.15) ^*∗∗*^	2.76 (1.11–4.41) ^*∗∗*^	1.12 (0.44–1.80) ^*∗∗*^
Model F	6.74^*∗∗∗∗*^	4.85^*∗∗∗*^	5.45^*∗∗∗∗*^
*R* ^2^	0.18	0.14	0.15

^*∗*^*ß*, (95% confidence interval),  ^*∗∗*^*p* < 0.01,  ^*∗∗∗*^*p* < 0.001, and  ^*∗∗∗∗*^*p* < 0.0001. HbA1c = hemoglobin A1c (in %, to convert to mmol/mol subtract 2.15 and multiply by 10.929); 90-day mean glucose expressed in mg/dL, to convert to mmol/L multiply by 0.0555).

## Data Availability

Data are available upon request from the corresponding author with approval from the University of Alabama at Birmingham Institutional Review Board. Please email Dr. Schmitt with request for data.
